# An Investigation of Genome-Wide Studies Reported Susceptibility Loci for Ulcerative Colitis Shows Limited Replication in North Indians

**DOI:** 10.1371/journal.pone.0016565

**Published:** 2011-01-31

**Authors:** Garima Juyal, Pushplata Prasad, Sabyasachi Senapati, Vandana Midha, Ajit Sood, Devendra Amre, Ramesh C. Juyal, Thelma BK

**Affiliations:** 1 Department of Genetics, University of Delhi, South Campus, New Delhi, India; 2 Dayanand Medical College and Hospital, Ludhiana, Punjab, India; 3 Department of Pediatrics, University of Montreal, Montreal, Canada; 4 Research Center, Sainte-Justine Hospital, Montreal, Canada; 5 National Institute of Immunology, New Delhi, India; Ohio State University Medical Center, United States of America

## Abstract

Genome-Wide Association studies (GWAS) of both Crohn's Disease (CD) and Ulcerative Colitis (UC) have unearthed over 40 risk conferring variants. Recently, a meta-analysis on UC revealed several loci, most of which were either previously associated with UC or CD susceptibility in populations of European origin. In this study, we attempted to replicate these findings in an ethnically distinct north Indian UC cohort. 648 UC cases and 850 controls were genotyped using Infinium Human 660W-quad. Out of 59 meta-analysis index SNPs, six were not in the SNP array used in the study. Of the remaining 53 SNPs, four were found monomorphic. Association (p<0.05) at 25 SNPs was observed, of which 15 were CD specific. Only five SNPs namely rs2395185 (*HLA-DRA*), rs3024505 (*IL10*), rs6426833 (*RNF186*), rs3763313 (*BTNL2*) and rs2066843 (*NOD2*) retained significance after Bonferroni correction. These results (i) reveal limited replication of Caucasian based meta-analysis results; (ii) reiterate overlapping molecular mechanism(s) in UC and CD; (iii) indicate differences in genetic architecture between populations; and (iv) suggest that resources such as HapMap need to be extended to cover diverse ethnic populations. They also suggest a systematic GWAS in this terrain may be insightful for identifying population specific IBD risk conferring loci and thus enable cross-ethnicity fine mapping of disease loci.

## Introduction

Ulcerative colitis (UC) and Crohn's disease (CD), the two sub-phenotypes of inflammatory bowel diseases (IBDs), are polygenic conditions that are suspected to result from dysregulated activation of immune mechansism to commensal microbes in genetically predisposed individuals. Considered to be a disease of the developed populations, there is growing evidence that the incidence of the disease may be high in developing countries as well. This is more so for ethnically heterogeneous populations such as the north Indian population, where we have recently shown that the incidence of disease in particular for UC is comparable to that reported in Western countries [1].

It is well established that genetic factors contribute to susceptibility for both CD and UC. Recently Genome wide association studies (GWAS) together with meta-analysis of GWAS findings involving UC [2]–[7] and CD [8]–[12] unearthed several risk conferring loci. Although some loci showed specific association with CD (*ATG16L1*) [10] or UC (*IL10*, *ECM1*, *HERC2*) [13], a substantial overlap in genetic risk factors between the phenotypes have also been observed with genes such as *IL23R* at the forefront [6], [14]–[15]. Discovery of these susceptibility genes, common as well as unique, has provided valuable insights into the link between the innate and adaptive immunity vis-à-vis risk for IBD.

Most candidate gene studies and recent GWAS have confirmed absence of associations with susceptibility variants in NOD2 gene and UC in Caucasians. However, we have previously reported notable allelic heterogeneity in this gene in a UC cohort from north India wherein the three frequently CD associated variants namely rs2066844, rs2066845 and rs2066847 were either absent or rarely present. Upon re-sequencing the gene in control subjects, only two reported polymorphisms, rs2066842 (Pro268Ser) and rs2067085 (Ser178Ser) were found. Of these, Pro268Ser that is common in Caucasians but associated with CD only in the presence of SNP13 was significantly associated with UC in our cohort. Analyzing the tag SNP profile for *NOD2* locus in this population revealed that the LD structure around Pro268Ser in the north Indians differs from that among Caucasians. These novel findings suggest population specific genetic profiles for UC in the north Indian population [16] warranting replication of other promising candidate genes.

With this background, we investigated whether the UC/CD genes/loci reported in the recent meta-analyses [6] were associated with UC in the ethnically distinct north Indian population.

## Results

Six of the 59 meta-analysis index SNPs were not present in the Infinium Human 660W-quad array used in this study (Table 1). Of the remaining 53 tested, four SNPs namely rs11465804 and rs11209026 (*IL23R*); rs2476601 (*PTPN22*) and rs4613763 (LOC730002) were monomorphic and therefore additional SNPs from these genes/loci were tested. Barring *IL23R*, both *PTPN22* and LOC730002 were not significant (p>0.05) in our cohort. The associated *IL23R* SNPs are shown in Table 2.

**Table 1:** Association status of 59 GWAS reported UC/CD specific susceptibility loci in a north Indian UC cohort.

Meta-analysis Index SNPs^a^	Chr	Gene/Locus of interest	Previously identified phenotype in caucasians	P-value	Allele	OR
rs1317209	1	RNF186	UC	0.62		
rs3806308	1	RNF186	UC	0.008*	A	0.80
rs6426833	1	RNF186	UC	0.0004**	G	0.74
rs2201841	1	IL23R	UC	0.044*	A	0.85
rs11465804^b^	1	IL23R	CD	-		
rs11209026^b^	1	IL23R	UC	-		
rs2476601^b^	1	PTPN22	CD	-		
rs2274910	1	ITLN1	CD	0.08		
rs10800309	1	FCGR2A	UC	0.40		
rs9286879	1	LOC441915	CD	0.15		
rs3024505	1	IL10	UC	0.001**	A	1.41
rs11584383	1	KIF21B	CD	0.41		
rs780094	2	GCKR	CD	0.027*	A	1.21
rs6706689	2	PUS10	UC	0.33		
rs13003464	2	PUS10	IBD	0.68		
rs917997	2	IL18RAP	CD	0.006*	A	1.23
rs3828309	2	ATG16L1	CD	0.06		
rs3197999	3	MST1	IBD	0.027*	A	1.21
rs4957048	5	CEP72	UC	0.65		
rs4613763^b^	5	LOC730002	CD	-		
rs2188962	5	LOC441108	CD	0.028*	A	1.27
rs10045431	5	IL12B	CD	0.33		
rs13361189^c^	5	IRGM	CD	-		
rs12529198	6	LYRM4	CD	0.020*	G	0.80
rs6908425	6	CDKAL1	CD	0.90		
rs3763313	6	BTNL2	CD	0.00002**	C	0.56
rs2395185	6	HLA-DRA	UC	0.000002**	A	0.63
rs7758080	6	MAP3K7IP2	CD	0.32		
rs2301436	6	FGFR1OP	CD	0.047*	A	1.17
rs7746082	6	PRDM1	CD	0.019*	A	1.24
rs17309827^c^	6	SLC22A23	CD	-		
rs1456893	7	ZPBP	CD	0.12		
rs4598195	7	DLD	UC	0.005*	C	0.78
rs1551398	8	TRIB1	CD	0.004*	A	1.24
rs10758669	9	JAK2	CD	0.008*	C	1.22
rs4077515	9	CARD9	UC	0.16		
rs4263839	9	TNFSF15	CD	0.019*	A	0.81
rs11190140	10	NKX2-3	IBD	0.006*	A	1.23
rs17582416	10	CUL2	CD	0.14		
rs10995271	10	ZNF365	CD	0.43		
rs7927894	11	C11orf29	CD	0.09		
rs1558744	12	LOC341333	UC	0.31		
rs971545	12	IL26	UC	0.73		
rs11175593^c^	12	LRRK2,MUC19	CD	-		
rs3764147	13	C13orf31	CD	0.282		
rs2066843	16	NOD2	CD	0.0002**	A	1.46
rs2066844^c^	16	CARD15	CD	-		
rs2066845^c^	16	CARD15	CD	-		
rs2066847^c^	16	CARD15	CD	-		
rs2872507	17	ZPBP2	CD	0.009*	A	1.22
rs2305480	17	GSDML	UC	0.011*	A	1.21
rs744166	17	STAT3	CD	0.047*	G	0.86
rs991804	17	CCL2	CD	0.06		
rs8098673	18	LOC728473	CD	0.038*	C	0.85
rs2542151	18	PTPN2	CD	0.041*	G	1.21
rs4807569	19	SBNO2	CD	0.53		
rs2836878	21	LOC391282	UC	0.011*	A	0.79
rs762421	21	ICOSLG	CD	0.09		
rs1736135	21	LOC388814	CD	0.85		

**^a^** **Mc Govern, et al., Genome-wide association identifies multiple ulcerative colitis susceptibility loci (2010) Nat Genet.; 42(4):332–7.**

**^b^** **Monomorphic.**

**^c^** **SNPs not in the Illumina Human600W-Quad used in this study.**

*p<0.05.

**Significant after Bonferroni correction.

**Table 2:** List of significant (p<0.05) SNPs in and around IL23R gene.

SNP	Allele	P-value	OR (95% Confidence Intervals)
rs10889657	G	0.005	0.79 (0.67–0.95)
rs1884444	C	0.01	0.83 (0.70–0.96)
rs2064689	A	0.01	0.81 (0.69–0.97)
rs10489630	C	0.02	0.83 (0.71–0.98)
rs1004819	G	0.04	0.86 (0.72–0.97)
rs7517847	C	0.004	0.79 (0.67–0.94)
rs6682033	G	0.01	0.76 (0.62–0.96)
rs1343151	A	0.01	0.77 (0.61–0.93)
rs11209032	G	0.02	0.84 (0.70–0.94)
rs1495965	A	0.03	0.85 (0.71–0.96)
rs3790562	G	0.02	0.69 (0.51–0.95)
rs3790565	G	0.01	0.76 (0.63–0.99)
rs4297265	G	0.04	0.86 (0.75–1.02)
rs2270614	A	0.04	0.86 (0.75–1.02)
rs7555183	A	0.02	0.84 (0.75–1.03)

**Figure 1:**
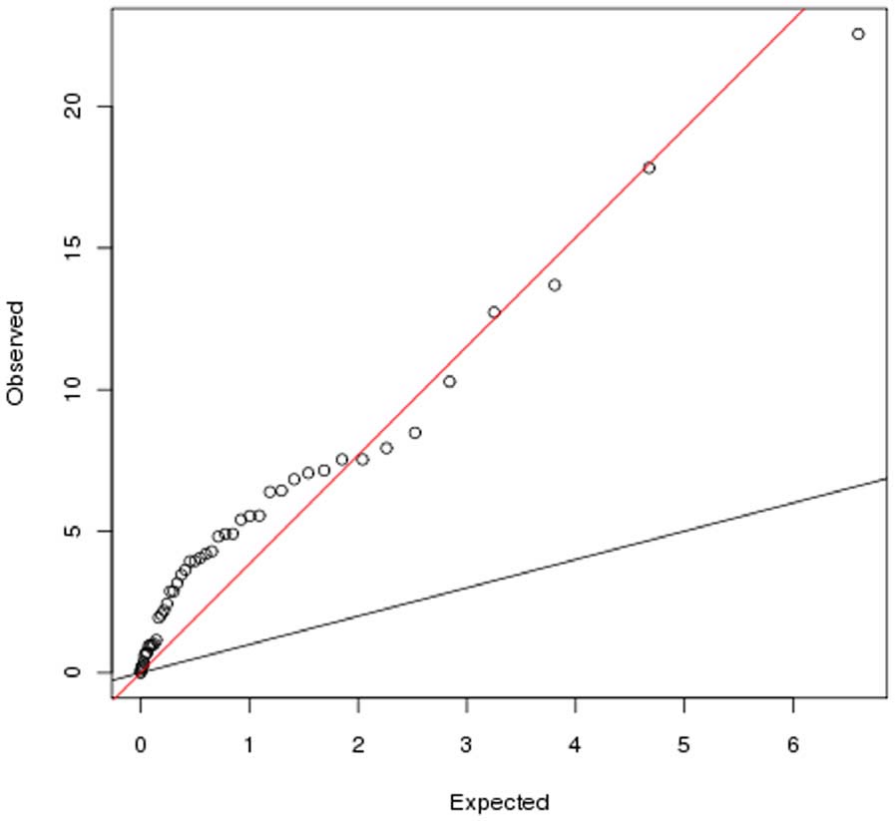
Quantile-Quantile plot of chi-square test of association p-values of 49 SNPs.

Of the remaining 49 index SNPs, associations were replicated with 25 SNPs (p<0.05) with 15 of them previously identified as CD specific (including rs2188962 located in IBD5 locus) (Table 1). Among these, five SNPs namely rs6426833 (*RNF186*, p = 0.0004), rs3024505 (IL10, p = 0.001), rs3763313 (*BTNL2*, p = 2.415e^−^05), rs2395185 (*HLA-DRA*, p = 2.038e^−^06) and rs2066843 (NOD2, p = 0.0002) withstood Bonferroni correction (Table 1, Fig. 1). It is noteworthy to mention here that we previously reported association of Pro268Ser in *NOD2*, a famous CD specific gene, [16] which is in complete LD (r^2^ = 1) with aforementioned SNP rs2066843.

While loci harbouring *ITLN1* and *CCL2*, both reported as CD specific genes showed borderline significance p = 0.07 and 0.06 respectively, other notable functional genes/ loci such as *CARD9*, *IL26*, *IL12B*, CEP72, *PUS10*, *FCGR2A*, *KIF21B*, *CDKAL1* and MAP3K7IP2 otherwise replicated in Caucasians were not significantly associated with UC in north Indians (Table 1). Interestingly, another promising CD associated candidate, *ATG16L1*, also showed modest association (p = 0.05) in our sample. With about 650 cases/850 controls and after accounting for 49 comparisons (alpha set at 0.001), the study had sufficient power (80% using QUANTO http://hydra.usc.edu) to detect associations with odds ratios 1.3 or higher (or 0.77 or lower) for allele frequencies between 20%∼30%, odds ratios 1.4 or higher (0.71 or lower) for allele frequencies between 10%–20% & odds ratios 1.6 or higher for allele frequencies of 5∼10% assuming a log-additive model of inheritance.

## Discussion

Recent GWAS have identified >30 susceptibility genes/loci that predispose populations of European origin to IBD. The credibility and relevance of these genetic association studies is indicated by the success of replication attempts in diverse ethnic groups. Thus, in this study we investigated the contribution of these IBD specific loci in our ethnically heterogeneous north Indian UC cohort in order to define its genetic architecture more conclusively.

Our study showed that SNPs from *IL23R*, *PTPN22* and LOC730002/*PTGER4* were largely monomorphic in our cohort. Though additional SNPs from in and around *PTPN22* and LOC730002 did not show any association with UC, findings from *IL23R* locus (Table 2) warrant discussion. *IL23R* is considered as a genuine “generic” IBD susceptibility gene and has attained genome-wide significance with both UC [4], [9], [18]–[20] and CD [9], [19], [21]–[22] in various GWAS and independent replication studies. Interestingly, the non-synonymous SNP (rs11209026), the most widely replicated marker, with a potential protective role in Caucasians [9], [19], [23] and rs11465804 were almost monomorphic in both UC cases and controls. However, significant association (p<0.05) of additional SNPs selected from both within and around this gene (Table 2) is strongly suggestive of *IL23R* being a potential susceptibility gene and therapeutic target for UC in the north Indian population as well. However, the strength of association of this gene may vary in different populations. It may be mentioned here that resequencing of the complete *IL23R* exonic regions in 30 north Indian population based controls did not reveal any exonic SNPs in this gene. Thus, the suggestive association of SNPs around this gene (Table 2) may indicate the role of regulatory variants in *IL23R* in UC etiology in our cohort. Alternatively, the associated SNP may be in linkage disequilibrium with another yet undetected causal variant. These results also demonstrate the importance of normative allelic data for populations under investigation while selecting SNPs for replication of association findings in them. Absence of *IL23R* SNPs (rs11465804 and rs11209026) has also been reported in Japanese, Korean and Chinese cohorts [7], [24]–[26]. Such a fluctuation in allele frequencies across geographic regions could be attributed to different environmental conditions leading to apparent genetic/allelic heterogeneity of disease between Asians and Caucasians.

An enticing highlight of this study is that we could replicate a few previously acknowledged UC specific SNPs in or near genes/ loci such as RNF186, IL10, DLD, and NKX2-3 with HLA-DRA leading the list (Table 1). The anti-inflammatory cytokine IL10 has long been proposed to limit intestinal inflammation, and genetically engineered IL-10 deficient mice develop spontaneous colitis suggesting it might serve as a therapeutic target for UC [27]. NKX2-3, association of which has previously been shown with CD, is a transcription factor gene found to be associated with UC among Caucasians [6] seems to be a generic IBD gene in our sample also. Reassessment of such potential regions in both Caucasian and north Indian populations, who are ethnically related to Caucasian stock [28] may illuminate the common key pathogenic pathways underlying UC.

It has been reported that there exists an excess clustering of both CD and UC in families, which underscores the concept that the genetic architecture of these two disorders are overlapping. Of the 49 informative index SNPs tested in our UC cohort, 17 have been previously reported to be CD specific (Table 1). Of these, observed association of functionally relevant CD loci such as JAK2, IL18RAP, LYRM4, TRIB1, TNFSF15, ZPBP2 with BTNL2 and NOD2 at the forefront is noteworthy (Table 1). Recent investigation has shown an association between BTNL2 gene and UC in population of European and Asian descent [29]–[30]. Both in our previous [16] and this study we observed association of *NOD2* with UC in the north Indian cohort suggesting the ethnic-specificity of this gene. Further, to investigate its possible contribution to CD, extensive resequencing in our CD cohort (N = 50) was carried out. Similar to UC, absence of SNPs 8, 12 and 13 and occurrence of Pro268Ser indicated that allelic heterogeneity with regards to *NOD2* may be at play for CD as well. It has been reported that SNPs 8, 12 and 13 represent 82% of the NOD2-mutated chromosomes, [31] and that these polymorphisms account for about 18% of the genetic risk of CD in the Caucasian population [32]. Thus, our findings reiterate population specific genetic susceptibilities underlying complex disorders such as IBD which is a pathogen driven condition. These observations were corroborated by *ATG16L1* (p = 0.05) (Table 1) further support that population specific disease susceptibility genes exist for IBD. Additionally, *FCGR2A-FCGR2C* region which reached genome-wide significance in both Japanese and Caucasian cohorts [6]–[7] was not significant in our population (Table 1). Similar findings have also been reported for TNFSF-15 wherein the variants strongly associated with Caucasian UC cohort were not significant in Japanese UC samples [33].

To summarize, our replication attempt of meta-analysis findings clearly reveal (a) partial concordance of Caucasian based meta-analysis results; and (b) apparent genetic/ allelic heterogeneity at UC/CD loci. It is likely that some SNPs that did not pass correction may be associated with UC in north Indians but the study did not have sufficient power to detect these associations. In conclusion, the observed disparity in the allele frequency of GWAS hits in our cohort confirms differences in genetic architecture between populations. These results also suggest that resources such as HapMap need to be extended to cover diverse ethnic populations within the Indian subcontinent in order to enhance their utility for the conduct of association studies within these heterogeneous populations. Further, as the current study was limited to a selection of SNPs identified as susceptibility markers from the recent UC specific meta-analysis, a systematic GWAS in this terrain may not only be insightful for identifying population specific IBD risk conferring loci but also enable cross-ethnicity fine mapping of disease loci. Collectively, these data may help define the genetic relationship between CD and UC and thus unravel common, as well as disease-specific mechanisms of pathogenesis in diverse populations.

## Materials and Methods

### Ethics Statement

Ethical approval for this study was given by the respective institutional ethical committees (IEC, DMCH and IEC, UDSC) and informed written consent was acquired from the participants.

### UC and control subjects

A case-control study was carried out in subjects recruited from a tertiary hospital in Punjab, India. In brief, the diagnosis of UC was based on standard criteria that included clinical, endoscopic, radiologic and histopathological criteria. Patients with infectious colitis and indeterminate colitis were excluded. Controls were individuals recruited from the same study hospital and included blood donors and patients diagnosed with other ailments not related to IBD. Controls were selected such that they were ethnically similar to the cases and whose age range (±10 years) was within that of the cases.

### DNA extraction and Genotyping

DNA was collected from peripheral blood samples of UC patients and control samples using conventional phenol-chloroform method. For replicating meta-analysis based associations, 648 cases and 850 controls were genotyped using Infinium Human 660W-quad. Quality control steps were applied before the SNP genotypes were included in the final analysis. The average genotyping success rate was 99% and no marker deviated significantly (P<0.0001) from Hardy–Weinberg equilibrium in controls. In addition, SNPs with a minor allele frequency (MAF)<0.05 and missingness rate >0.05 were excluded. SNPs were tested for association with UC by Chi-square test implemented in PLINK [16] and Bonferroni correction was also applied.

### Resequencing

Exonic and exon-intron boundary regions of both *NOD2* and *IL23R* were amplified by PCR and sequenced on an ABI 3730 genetic analyzer. Details of primers used for amplification of all the exons are available on request.
